# Spontaneous mode non-invasive ventilation fails to treat respiratory failure in a patient with Multi-mincore disease: a case report

**DOI:** 10.1186/1757-1626-1-93

**Published:** 2008-08-15

**Authors:** Krishna B Sriram, Andrew Thornton, Ral Antic

**Affiliations:** 1 Sleep Disorders Service, Department of Thoracic Medicine, Royal Adelaide Hospital, Adelaide, Australia

## Abstract

The increased morbidity and mortality resulting from respiratory failure in patients with neuromuscular disorders and/or kyphoscoliosis can be reversed with non-invasive ventilation. Spontaneous mode bilevel pressure ventilation is preferred to other modes of ventilation, due to relative ease of use, but may not be suitable for all patients. We report a 27-year old woman with Multi-minicore disease whose respiratory failure was refractory to spontaneous mode bilevel pressure ventilation. When we altered settings and provided mandatory inspiratory rise time and respiratory rate, it augmented her respiratory efforts and improved ventilation. Our case report describes the benefit of individualising non-invasive ventilation in the management of respiratory failure due to neuromuscular weakness and kyphoscoliosis.

## Introduction

Chronic respiratory failure develops in patients with restrictive ventilatory disorders due to a combination of decrease in inspiratory efforts, reduced minute ventilation and hypoventilation during sleep [1,2].

NIV is recommended in these patients as it improves pulmonary mechanics, oxygen saturation, sleep parameters and quality of life [2,3]. It is important to titrate NIV parameters to individual patients and not rely on default settings, otherwise therapeutic failure will ensue. We report a patient with Multi-minicore disease, a rare disorder characterized by neuromuscular weakness and kyphoscoliosis with respiratory failure unresponsive to spontaneous mode bilevel pressure ventilation.

## Case presentation

A 27-year old woman with Multi-minicore disease presented with severe daytime somnolence (ESS 19) and headaches. Physical examination revealed pectus carinatum and kyphoscoliosis. Spirometry showed FEV1 of 0.73 L (25% predicted), FVC of 0.77 L (23% predicted) and FEV1/FVC of 95%. Maximal expiratory and inspiratory pressures were 17% and 30% predicted respectively. Computed tomography of chest did not show any pulmonary abnormality. Diagnostic PSG showed frequent obstructive apneas, hypoventilation and oxygen desaturation (Table 1).

**Table 1:** Arterial blood gas and Polysomnography Parameters: Baseline vs. S-mode NIV vs. ST mode NIV

	**Baseline**	**Spontaneous (S) ** **mode BiPAP**	**Spontaneous Timed ** **(ST) mode BiPAP**
PaO2	45	57	80.6
PaCO2	83.4	73.5	55
PH	7.35	7.35	7.36
≥ 3% O2 desaturation/hr	149	57	20.7
Peak TcCO2 – total sleep time	88	88	68

**Figure 1:**
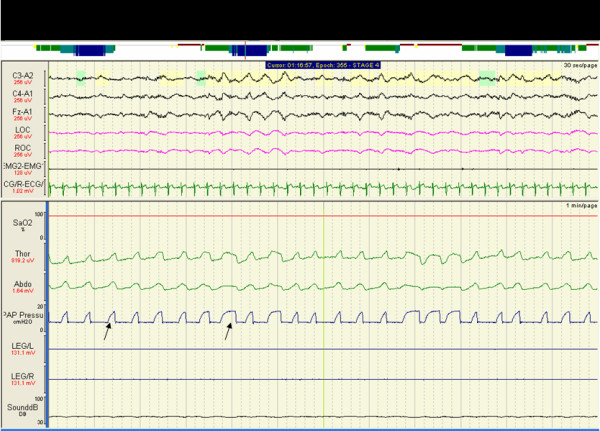
Polysomnogram on S-mode BiPAP showing irregular nasal flow (CPAP Pressure) due to patient-ventilator dyssynchrony (see arrows).

Spontaneous (S) mode bilevel pressure ventilation (BiPAP ^® ^Duet LX System, Respironics^®^, Pennsylvania, USA) was commenced and a full face mask (Ultra Mirage™ ResMed Ltd, Australia) was applied to minimize leak. PSG on S-mode BiPAP and daytime ABG analysis was performed 7 days after commencement of therapy. There was minimal improvement in respiratory indices (Table 1) and PSG revealed premature inspiration-expiration cycling (Figure 1).

**Figure 2:**
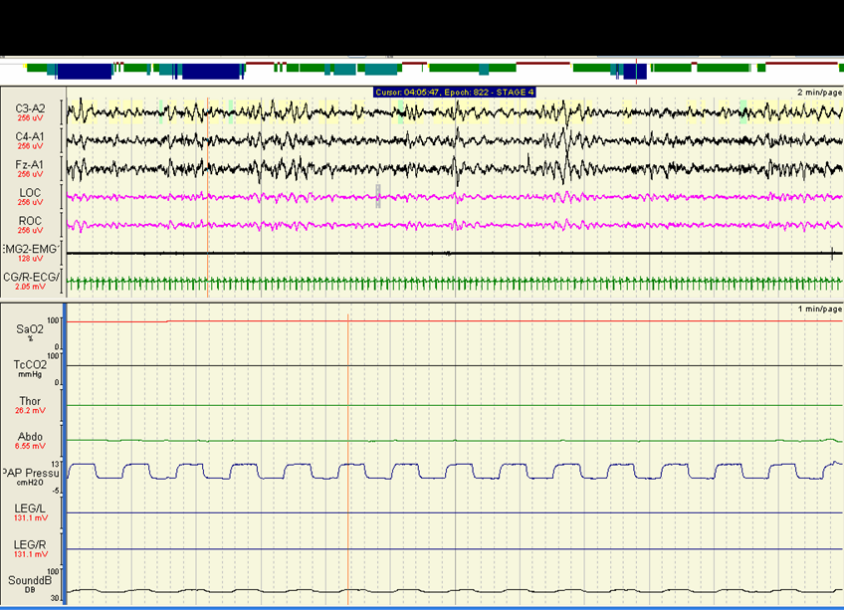
Polysomnogram on ST-mode BiPAP showing regular nasal flow (CPAP pressure) and correction of patient-ventilator dyssynchrony.

Subsequently spontaneous timed (ST) mode BiPAP was initiated (BiPAP ^® ^Harmony™) with a minimum inspiratory time of 2 sec and respiratory rate of 12 breaths per minute. PSG on ST-mode BiPAP and daytime ABG analysis was checked after 7 days of therapy. On these settings > 90% of breaths were assisted with ST mode BiPAP (Figure 2) with an improvement in respiratory indices (Table 1), resolution of daytime somnolence (ESS – 8) and headaches.

## Discussion

Multi-minicore disease, an autosomal recessive disorder, is characterized by axial muscle weakness, kyphoscoliosis and respiratory impairment. Many patients progress to respiratory failure and require NIV [4]. However there are no recommendations on what mode of NIV or parameters best treats the respiratory failure due to Multi-minicore disease.

NIV is broadly categorised into volume controlled and pressure controlled (spontaneous (S) mode, spontaneous timed (ST) mode or timed (T) mode) ventilation [5,6]. There has been a progressive shift from volume controlled to bilevel pressure controlled ventilators, especially spontaneous mode ventilators, because the latter are smaller, quieter, easier to use and cheaper [6]. However spontaneous mode on default settings may not be suitable for all patients with respiratory failure [5,7].

In patients with neuromuscular weakness or kyphoscoliosis bilevel pressure controlled ventilators on default settings may result in premature inspiration-expiration cycling [5], resulting in persistence of insufficient inspiratory efforts, reduced tidal volume and hypoventilation during sleep. Such problems can be attenuated when the cycling is tailored to the patient's respiratory mechanics.

When we identified that our patient's respiratory failure was refractory to spontaneous mode ventilation, we provided mandatory inspiratory rise time and respiratory rate which increased her respiratory effort resulting in improvements in oxygenation and reduction in hypercapnia.

Our report highlights the importance of individualising NIV treatment when treating respiratory, especially in with neuromuscular weakness and kyphoscoliosis.

## List of abbreviations

NIV: Non-invasive ventilation; PSG: Polysomnography; S-mode BiPAP: Spontaneous mode bilevel positive airway pressure; ST-mode BiPAP: Spontaneous Timed mode bilevel positive airway pressure; FEV1: Forced expiratory volume in 1 second; FVC: Forced vital capacity; ABG: Arterial blood gas; TcCO2: Transcutaneous carbon dioxide; REM: Rapid eye movement; NREM: Non-rapid eye movement; ESS: Epworth sleepiness score.

## Competing interests

The authors declare that they have no competing interests.

## Authors' contributions

KBS reviewed the case notes and prepared the manuscript. KBS, AT and RA read and approved the final manuscript.

## Consent

Written informed consent was obtained from the patient for publication of this case report and accompanying images. A copy of the written consent is available for review by the Editor-in-Chief of this journal.
